# Resting EEG Microstates and Autonomic Heart Rate Variability Do Not Return to Baseline One Hour After a Submaximal Exercise

**DOI:** 10.3389/fnins.2018.00460

**Published:** 2018-07-10

**Authors:** Jérôme N. Spring, Nicolas Bourdillon, Jérôme Barral

**Affiliations:** ^1^Institute of Sport Sciences, Faculty of Social and Political Sciences, University of Lausanne, Lausanne, Switzerland; ^2^Institute of Sport Sciences, Faculty of Biology and Medicine, University of Lausanne, Lausanne, Switzerland

**Keywords:** EEG, microstates, HRV, exercise, recovery

## Abstract

Recent findings suggest that an acute physical exercise modulates the temporal features of the EEG resting microstates, especially the microstate map C duration and relative time coverage. Microstate map C has been associated with the salience resting state network, which is mainly structured around the insula and cingulate, two brain nodes that mediate cardiovascular arousal and interoceptive awareness. Heart rate variability (HRV) is dependent on the autonomic balance; specifically, an increase in the sympathetic (or decrease in the parasympathetic) tone will decrease variability while a decrease in the sympathetic (or increase in the parasympathetic) tone will increase variability. Relying on the functional interaction between the autonomic cardiovascular activity and the salience network, this study aims to investigate the effect of exercise on the resting microstate and the possible interplay with this autonomic cardiovascular recovery after a single bout of endurance exercise. Thirty-eight young adults performed a 25-min constant-load cycling exercise at an intensity that was subjectively perceived as “hard.” The microstate temporal features and conventional time and frequency domain HRV parameters were obtained at rest for 5 min before exercise and at 5, 15, 30, 45, and 60 min after exercise. Compared to the baseline, all HRV parameters were changed 5 min after exercise cessation. The mean durations of microstate B and C, and the frequency of occurrence of microstate D were also changed immediately after exercise. A long-lasting effect was found for almost all HRV parameters and for the duration of microstate C during the hour following exercise, indicating an uncompleted recovery of the autonomic cardiovascular system and the resting microstate. The implication of an exercise-induced afferent neural traffic is discussed as a potential modulator of both the autonomic regulation of heart rate and the resting EEG microstate.

## Introduction

At rest, the brain continually integrates information from external and internal sources, resulting in rapid changes in the distribution of neural activation at the cortical and sub-cortical levels (Michel et al., 2009). The summation of this electrical activity propagates to the scalp surface, resulting in specific map topographies. Assuming that different map topographies are generated by different neuronal configurations, resting microstate analysis enables the quantification over time of spontaneous brain activity from the surface electric fields (Michel et al., 2009; Brunet et al., 2011). Conventional multichannel electroencephalographic (EEG) recordings are segmented into a sequence of discrete map topographies with archetypal and reproducible temporal properties (Koenig et al., 2002; Lehmann et al., 2009; Khanna et al., 2015). Four map topographies (A, B, C, and D) can be extracted from an EEG signal, and explain approximately 70–80% of the total topographic variance (Khanna et al., 2015). Each of these maps has been associated with a group of interconnected brain nodes that are organized into a network and active at rest (Britz et al., 2010). Map A has been associated with the visual resting state network (RSN), map B with the auditory RSN, map C with the salience RSN, and map D with the attentional RSN (Britz et al., 2010).

A single session of dynamic whole-body exercise modulates microstate temporal features (Spring et al., 2017). Specifically, duration and relative time coverage of map C significantly increased following a 30-min cycling exercise in trained participants. The predominance of microstate C was supported by changes in the microstate syntax, where the propensity to transit toward map C was greater after exercise. The authors postulated that post-exercise microstate might be related to an afferent signaling pathway via projections to the salience RSN (Spring et al., 2017). Indeed, the salience network integrates information such as pain and muscular sensation (Craig, 2003; Singer et al., 2009) and correlation exists between peripheral muscular alterations and the increase in microstate map C mean duration (Spring et al., 2017). However, the salience network also responds to internal signals coming from autonomic processes and mediates cardiovascular arousal (Critchley et al., 2000; Bechara and Naqvi, 2004; Pollatos et al., 2007; Eckert et al., 2009; Menon, 2015). The present study aims to confirm the implication of an afferent activity modulation in post-exercise microstate changes by evaluating the autonomic cardiovascular activity and its recovery. The potential links between the EEG microstates and the cardiovascular control will be observed throughout the recovery kinetic of both variables.

The specificity of large-muscle group exercise is the massive cardiovascular response that copes with the increase in the metabolic requirement. The optimal physiological balance in the body and the successful cardiovascular control of blood pressure and distribution of blood flow is under the control of the parasympathetic and sympathetic autonomic nervous system (Christensen and Galbo, 1983; Murphy et al., 2011). At exercise onset, parasympathetic withdrawal drives the sudden increase in heart rate (HR). Increase in the sympathetic tone drives the subsequent increase in HR (Tulppo et al., 1996). At cessation of exercise, parasympathetic reactivation principally determines the fall in HR (Perini et al., 1989; Coote, 2010). HR then decreases slowly in an exponential manner (Savin et al., 1982), depending on different factors such as the level of fitness for instance (Darr et al., 1988). Depending on the exercise intensity and duration, this autonomic reactivation can last several hours (Furlan et al., 1993; Mourot et al., 2004; Seiler et al., 2007; Stanley et al., 2013; Perkins et al., 2017) and seems to reflect the time required for a set of physiological and metabolic parameters to return to homeostasis (Stanley et al., 2013). The sympathetic and parasympathetic components can be indirectly assessed using the heart rate variability (HRV) obtained from a conventional electrocardiogram (ECG) recording. HRV is defined as the variability between beat-to-beat (R-R) intervals and reflects the autonomic background of the fluctuation in HR (Malik, 1996). Standards from the task force of the European Society of Cardiology more precisely differentiate the relative participation of the sympathetic and parasympathetic components (Malik, 1996). Accordingly, the root mean square of the successive differences between R-R intervals (RMSSD) reflects the rapid fluctuations in parasympathetic activity. The high-frequency power (HF: 0.15–0.40 Hz) is modulated by respiration-induced variations in HR and reflects mainly parasympathetic activity. The low-frequency power (LF: 0.04–0.15 Hz) reflects both the sympathetic and parasympathetic activities. Consequently, it is assumed that the LF/HF ratio provides information about the sympatho-vagal balance (Malik, 1996).

The salience network is a connected large-scale network anchored in the anterior insula (AI) and dorsal anterior cingulate (dACC) cortex (Menon and Uddin, 2010; Menon, 2015). The cardiac autonomic activity involves cardiovascular control centers within the brain stem, but also cortical and subcortical structures (Craig, 1995, 2002, 2003; Menon and Uddin, 2010). For instance, the insula and cingulate cortices have been shown to play an important role in cortical regulation of autonomic cardiac activity (Williamson et al., 2003; Sander et al., 2010; Macefield and Henderson, 2015; Williamson, 2015). Using functional magnetic resonance imaging method, Sander et al. (2010) showed an increase in neurovascular coupling in the sensorimotor and insular cortices, and a decrease in the midcingulate cortex during static handgrip exercise. More specifically, neuroimaging studies have associated HRV with fluctuations in brain connectivity within different neural structures including the dACC and AI (Critchley et al., 2003; Napadow et al., 2008; Lane et al., 2009; Thayer et al., 2012; Chang et al., 2013). Together, these studies confirm the implication of the salience network in the autonomic cardiac regulation, further supporting a putative link between the autonomic cardiovascular activity and the post-exercise microstate C changes.

The present study aimed to further explore the implication of map C-related salience RSN after exercise and the possible association with the autonomic cardiovascular response. The objective was to describe the microstates and HRV time-courses after a single-bout of endurance exercise and during the 60-min recovery period in non-athlete males and females. We predicted that the exercise-induced modulation of map C would persist as long as the autonomic HR modulation had not returned to baseline.

## Materials and Methods

### Participants

The forty-two healthy right-handed volunteers enrolled in this study completed the French version of the Physical Activity Readiness Questionnaire (PAR-Q) to exclude obvious risk factors for adverse cardiac events during exercise. The exclusion criteria included recent musculoskeletal pathology that may interfere with exercise; a personal history of psychiatric, neurological or cardiovascular disorders; alcohol consumption above 3 units per day; asthma; and current drug usage. All of the volunteers completed the Baecke questionnaire (Baecke et al., 1982) for the evaluation of habitual physical activity using a score between 1 and 5 for sport, work, and leisure activities, and also a total score obtained from the sum of the 3 previous domains. Before starting the protocol, all participants provided written informed consent. The study was approved by the local ethic committee (CER-VD: 2016-01730) and conducted in compliance with the applicable legal requirements.

### Experimental Protocol

According to the literature, an exercise duration of approximately one half hour at an intensity around the anaerobic threshold is enough to induce HRV changes for several minutes after exercise cessation (Terziotti et al., 2001; Seiler et al., 2007). Therefore, the exercise consisted of a 25-min exercise on a cycle-ergometer (Lode Excalibur Sport, Groningen, Netherlands) at a subjective intensity of 15 on the Borg scale of 6–20. The intensity was individualized based on the subjective effort perception, assuring that exercise results in a similar relative physiological response between subjects. A score of 15 corresponds to a perceived physical effort considered as “hard” and is thought to be close to the ventilatory threshold (Purvis and Cukiton, 1981; Hill et al., 1987). The exercise started with an incremental protocol starting at 60 watts with an increase of 15 watts every minute. At the end of each step, subjects were asked to provide a score between 6 (no exertion) and 20 (maximal exertion) on the 6–20 Borg scale in order to quantify the rate of perceived exertion (RPE). When a score of 15 was reached, the increment in workload ceased, and the 25-min constant-load exercise began. The time required to reach 15 was considered as the warm-up period. If RPE deviated by more than ±1 unit during the first 5 min of constant-load cycling, the load was adjusted (increased or decreased) to ensure a stable perceived effort during the entire 25 min. The experimental protocol lasted 3 h. Participants were asked to avoid severe exercise 24 h before the test. Alcohol and caffeine consumption were proscribed for the 12 h preceding the test and water was provided *ad libitum* during the protocol.

### EEG and ECG Data Acquisition

An EEG and an ECG were simultaneously recorded in light-shielded room for 5 min before (BSL) and 5 min after the exercise session (P05), as well as 15 (P15), 30 (P30), 45 (P45), and 60 (P60) minutes after the end of exercise. Participants were comfortably seated on a chair with their chin rested on a chin support to reduce head movement artifacts. The only instruction given was to close the eyes and relax during the recording. The EEG was collected using a 64-chanel BioSemi Active two amplifier system (Biosemi, Amsterdam, Netherlands) mounted according to the International 10–20 recommendations. The ECG was collected using two flat-type electrodes (BiomSemi, Amsterdam, Netherlands) placed under the right clavicle on the midclavicular line, and near the 5th left intercostal space on the anterior axillary line. Both signals were referenced to the active Common Mode Sense-Driven Right Leg (CMD-DRL) ground system incorporated in the BioSemi device. EEG and ECG data were simultaneously collected at a sampling frequency of 2048 Hz, and the EEG electrode impedance was kept below 10 kΩ. The experiment was conducted in a cool air-conditioned room (temperature: 19°C, humidity: 30%, CO_2:_ 550 ppm), and a breathable Lycra cap (BiomSemi, Amsterdam, Netherlands) was used to help dissipate heat. A fan was also placed in front of the subject during the exercise protocol to ensure minimal artifacts of sweat on the collected signals.

### EEG and ECG Data Processing

The EEG analyses were performed using the Cartool software^1^ and ECG analyses were processed using custom MATLAB scripts (version 8.5.0, MathWorks, Natick, MA, United States). The ECG signal was down-sampled at 256 Hz and ECG R peaks were identified from the ECG using an automatic extrema detection method. Ectopic beats were compensated using means of interpolation to calculate normal-to-normal intervals (R-R). From this signal, the HRV parameters were obtained. In the time domain, we computed the mean HR during the 5-min recording (mean HR) and the root mean square of the successive differences between R-R intervals (RMSSD). For the analysis in the frequency domain, the following parameters were extracted: the spectral power in the low-frequency (LF, 0.04–0.15 Hz) and high-frequency bands (HF, 0.15–0.40 Hz) in ms^2^, the values for LF and HF expressed in normalized units (normalized to the total power) and labeled nLF and nHF; and the LF/HF ratio. The spectral power was estimated using a Fast Fourier Transform on the resampled RR intervals (4 Hz) using a window length of 250 data points and an overlap of 50%. Non-stationarities may influence and distort HRV indices (Magagnin et al., 2011). Accordingly, in order to estimate the fraction of non-stationarity in the HRV recordings, the Likelihood ratio test was performed on HRV data.

The EEG signals were band-pass filtered between 1 Hz and 40 Hz to exclude unwanted slow wave activities generated by sweating and skin potentials and also to avoid high frequency muscular artifacts and non-cortical electrical sources. The data were visually inspected, and an infomax-based independent component analysis (ICA) was applied. Based on the waveform, the time course, and the topography of each ICA component (Jung et al., 2000), residual eye-twitching and cardiac artifacts were removed. The reconstructed signal was down-sampled to the same sampling rate as for the ECG (256 Hz), bad electrodes were interpolated with a 3-D spherical spline, and the signal was recomputed to the common average reference. The microstate analysis followed a conventional procedure applied in previous studies (Britz et al., 2010; Brunet et al., 2011; Tomescu et al., 2014; Spring et al., 2017) and implemented in the Cartool software (Brunet et al., 2011; Michel et al., 2009). To summarized, the pre-processed datasets collected in BSL, P05, P15, P30, P45, and P60 were used to obtain the 4 expected convention microstate templates. The topographies at Global Filed Power (GFP) peaks were submitted to a k-means clustering to identify for each participant in each condition the four maps that maximally explained the topographic variance. The GFP is a reference-free measure of total filed strength and corresponds to the standard deviation of the potential field (Skrandies, 1990). After applying the clustering to individuals, a second clustering was applied using the 4 best individual topographies to obtain the best representative microstate map for the group across the 6 periods of measurement. Based on multiple criteria selection, the software automatically generated the best cluster for the group, which corresponded to the expected 4 maps. These maps were spatially similar to those described in previous studies (Michel and Koenig, 2017) and thus were labeled as map A to D (Koenig et al., 1999). Finally, individual pre-processed EEG recordings were allocated to one of these 4 maps based on their spatial correlation. This back-fitting process allows to compute for each microstate the mean continuous period of time assigned to a given microstate (mean duration), the number of time that a map occurred in one second (frequency of occurrence), the relative percentage of time covered by a map (time coverage), and the probability of transition from one map to another (microstate syntax). In the present study, the microstate syntax was considered as Markov transition matrix with a finite number of states undergoing transitions from one to another (i.e., Markov chain) (Lehmann et al., 2005; Gärtner et al., 2015). The probability of transition from a given map to another was obtained by calculating the occurrence frequency of transitions from this map to all others. In order to make the probability of transition changes independent from modifications in the microstate occurrence, each pair of transition was corrected from randomness by subtracting the expected probability from the observed ones as previously used by Spring et al. (2017).

A possible association between microstate and HRV parameters changes was tested as follows. First, to identify if microstate and HRV responses were associated with exercise, we computed the difference between BSL and P05 (ΔBSL-P05) for microstate and HRV parameters before using these differences as correlation variables. Second, to explore a putative association during the post-exercise recovery, we proceeded in similar manner by computing the difference between P05 and P60 (ΔP05-P60). Finally, we computed an index of global recovery, which corresponds the difference between BSL and P60 (ΔBSL-P05).

### Statistical Analyses

Statistical analyses were performed with Statistica 12.6 software (Statsoft, Tulsa, OK, United States). Normality of each variable was evaluated by Shapiro–Wilk tests and significance was set at *p* < 0.05. The data are presented as the mean ± standard deviation (*SD*) in the text and the mean ± 95% confidence interval (CI) in the figures. One way-way repeated-measure ANOVA (rmANOVA) with factor Time were used to compare the HRV variables between the different times of measurement (BSL, P05, P15, P30, P45, and P60) and Bonferroni *post hoc* tests were applied for significant interactions. Friedman ANOVAs with follow-up Wilcoxon sign rank tests were used in a few cases in which conditions for using parametric tests were not reached. We compared the 4 microstate maps changes across time using two-way rmANOVA 6(*TIME*) × 4(*MAP*). These analyses were conducted for the microstate mean duration, time coverage, and frequency of occurrence. The microstate syntax was investigated using a two-way rmANOVA 6(*TIME*) × 12(*PAIRS*) to identify a difference between the 12 pairs of transition across BSL, P05, P15, P30, P45, and P60. This rmANOVA was also performed on the microstate syntax corrected for occurrence. When two-way rmANOVA revealed significant interactions, pairwise contrast were performed using the Bonferroni correction. The presumed association between microstates map C and HRV parameters changes were explored using Pearson correlations on the absolute difference between BSL and P05 (ΔBSL-P05), P05 and P60 (ΔP05-P60), and BSL and P60 (ΔBSL-P60).

## Results

### Participants

Forty-two volunteers completed the protocol, and four participants were excluded from analysis. Two subjects had artifacts due to important eyelid twitching or involuntary movements, and two subjects were anxious because of personal reasons. The remaining 38 volunteers (22 females and 16 males) were 24 ± 4 years old. Their average height, body mass, and body mass index were 173 ± 9 cm, 68 ± 13 kg and 22 ± 3 kg⋅m^-2^, respectively. Participants were physically active as demonstrated by a total Baecke score of 8.3 ± 1.5, with a value of 2.1 ± 0.7 for work, 3.2 ± 0.9 for sport, and 2.9 ± 0.6 for leisure.

### Exercise Data

The warm-up period, which corresponded to the time required for reaching 15 on the Borg scale of 6–20 was 7 ± 2 min. The total duration of exercise was 32 ± 2 min, the mean HR during the 25 min of constant-load exercise was 160 ± 19 bpm, and the mean RPE was 15.1 ± 0.6. The HR and RPE time courses during the cycling exercise are shown in **Table 1**.

**Table 1:** Heart rate and Borg rating of perceived exertion scale (6–20) during the 25-min cycling exercise.

	BSL	Exercise duration (min)				
		5	10	15	20	25
	Mean ± SD	Mean ± SD	Mean ± SD	Mean ± SD	Mean ± SD	Mean ± SD
HR (bpm)	66 ± 11	155 ± 20	159 ± 20	159 ± 20	163 ± 18	164 ± 18
Borg scale	− ± −	15.0 ± 0.7	15.0 ± 0.8	15.1 ± 1	15.1 ± 0.8	15.3 ± 0.9

*BSL, baseline; HR, heart rate; SD, standard deviation.*

### HRV Data

**FIGURE 1:**
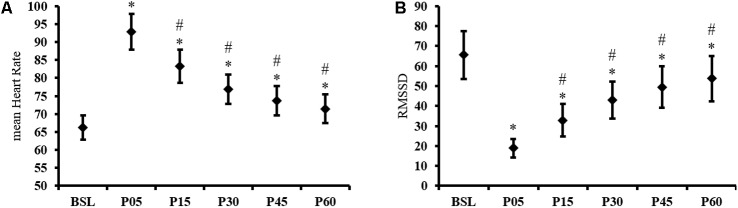
Time domain HRV results. Time course of **(A)** mean Heart Rate and **(B)** RMSSD, recorded at baseline (BSL) and 5 (P05), 15 (P15), 30 (P30), 45 (P45), and 60 (P60) minutes after exercise cessation. Error bars indicate the 95% confidence interval. ^∗^Indicates a significant difference from BSL. ^#^Indicates a significant difference from P05; *p*-value < 0.05.

The endurance exercise resulted in a significant *TIME* effect for mean HR [*F*(5,185) = 194.35, *p* < 0.001]. In BSL, the mean HR was 66 ± 11 bpm and increased significantly by 41 ± 15% immediately after the end of exercise in P05 (*p* < 0.001). Compared to P05, the mean HR decreased by 10 ± 4%, 17 ± 5%, 21 ± 6%, and 23 ± 6% in P15, P30, P45, and P60, respectively (all *p* < 0.001). In P60, the HR remained significantly higher than BSL (8 ± 7%, *p* < 0.001), indicating an incomplete recovery (**Figure 1A**). The Friedman ANOVA revealed a significant effect of *TIME* for RMSSD [χ^2^ (*N* = 38, *d* = 5) = 119.12, *p* < 0.001]. Compared to BSL, RMSSD was reduced to 67 ± 27% after exercise in P05 (*p* < 0.001). During the post-exercise recovery period, RMSSD was different from P05 in P15, P30, P45, and P60 (all *p* ≤ 0.001) and remained different from BSL in P60 (*p* < 0.001) (**Figure 1B**).

**FIGURE 2:**
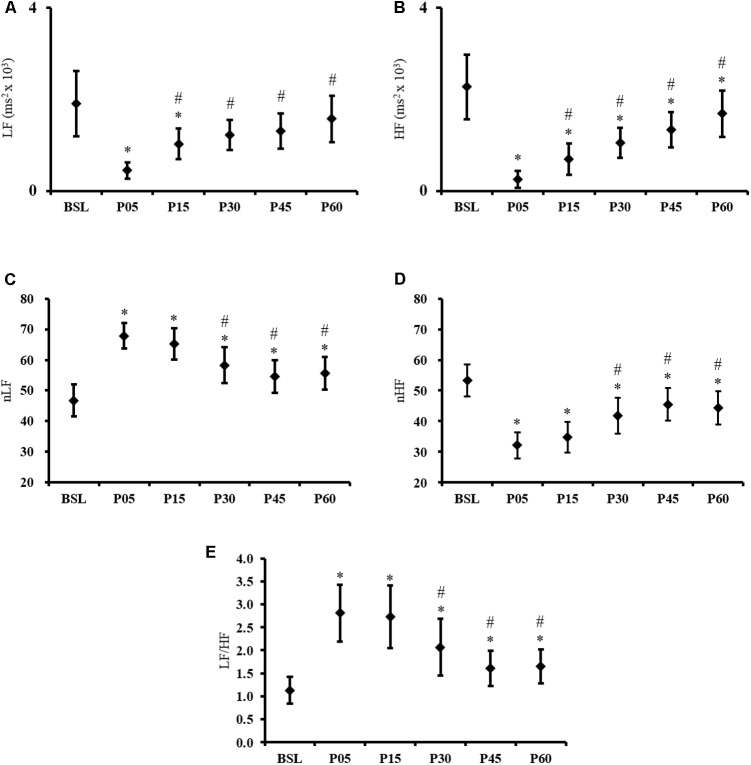
Frequency domain HRV results. Time course of **(A)** LF, **(B)** HF, **(C)** nLF, **(D)** nHF, and **(E)** LF/HF ratio, recorded at baseline (BSL) and 5 (P05), 15 (P15), 30 (P30), 45 (P45), and 60 (P60) minutes after exercise cessation. Error bars indicate the 95% confidence interval. ^∗^Indicates a significant difference from BSL. ^#^Indicates a significant difference from P05; *p*-value < 0.05.

The Friedman ANOVA revealed a significant effect of *TIME* for LF [χ^2^ (*N* = 38, *d* = 5) = 58.4, *p* < 0.001] and HF [χ^2^ (*N* = 38, *d* = 5) = 11.6, *p* < 0.001]. Compared to BSL, the follow-up Wilcoxon signed rank tests revealed a significant difference with P05 (*p* < 0.001) and P15 (*p* = 0.008) for LF; and with P05 (*p* < 0.001), P15 (*p* < 0.001), P30 (*p* < 0.001), P45 (*p* < 0.001) and P60 (*p* = 0.002) for HF. Compared to the P05 condition, the P15, P30, P45, and P60 condition were significantly different for LF and HF (all *p* < 0.001) (**Figures 2A,B**). Although the LF and HF expressed in normalized unit are dependent markers of autonomic cardiovascular activity, the results of both variables were reported to provide a global vision of HRV changes. The frequency-domain HRV analysis revealed a *TIME* effect for the nLF and nHF [*F*(5,185) = 25.44, all *p* < 0.001]. Compared to BSL, nLF and nHF were altered after exercise in P05 and remained different from BSL across all time points measured (all *p* < 0.001). In P30, P45, and P60, the nLF and nHF were significantly lower than in P05 (all *p* < 0.001) (**Figures 2C,D**). A significant *TIME* effect was also found for the LF/HF ratio [χ^2^ (*N* = 38, *d* = 5) = 65.45, *p* < 0.001]. After a significant increase induced by exercise (from 1.13 ± 0.9 in BSL to 2.8 ± 1.9 in P05, *p* < 0.001), the ratio remained around a similar value in P15 (2.7 ± 2.1), and decreased gradually during the recovery period without returning to baseline (all *p* ≤ 0.003) (**Figure 2E**). The Likelihood ratio tests of stationarity performed on HRV data indicate that 16, 8, 18, 31, 24, and 18% of the data were non-stationary in BSL, P05 P15, P30, P45, and P60 condition respectively.

### EEG Data

**FIGURE 3:**
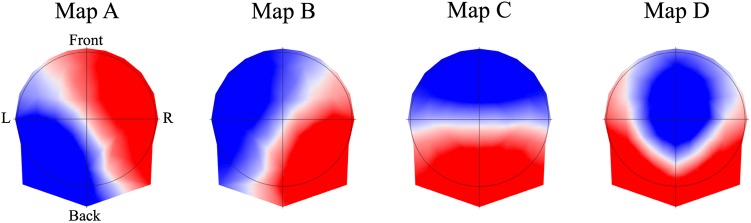
The four resting EEG microstate topographies. The maps obtained across condition were labeled in class A, B, C, and D, according to previous studies. Note that EEG microstate analysis ignores the topographic polarity.

The 4 best representative topographies of all individuals across conditions explained 85% of the total variance and were labeled as map A, B, C and D (**Figure 3**).

**FIGURE 4:**
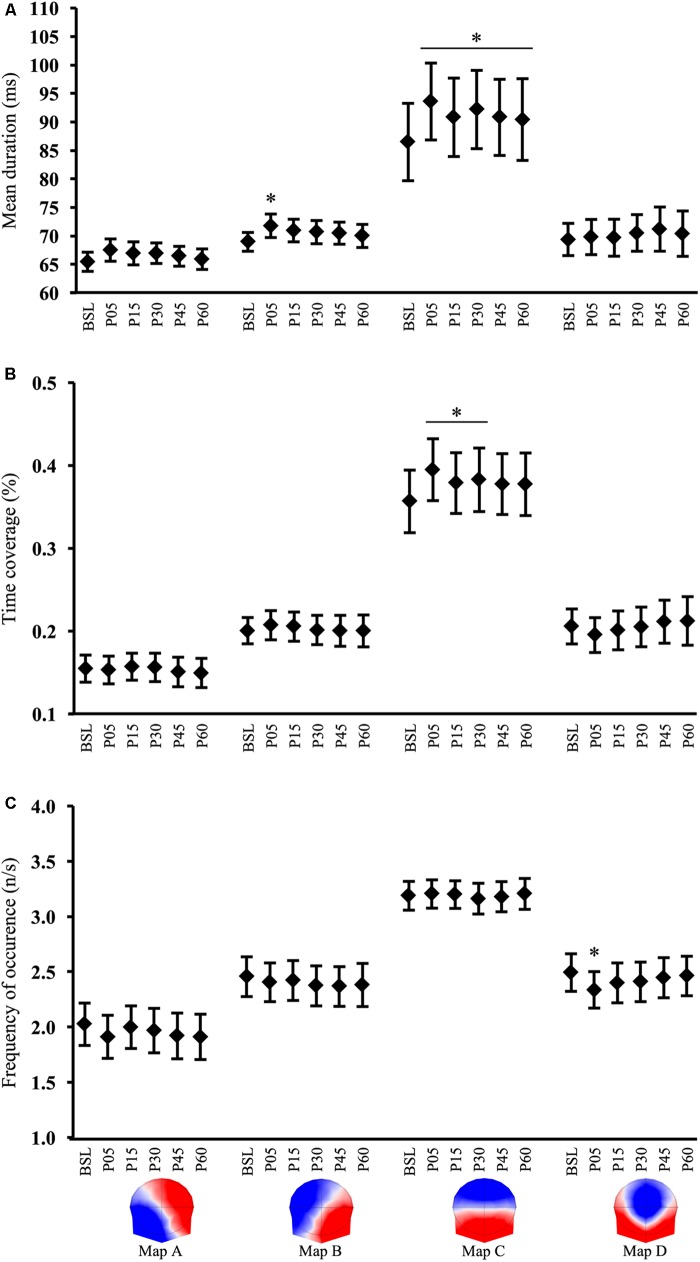
Microstate analysis results. Time course of microstates **(A)** mean duration, **(B)** time coverage, and **(C)** frequency of occurrence, computed in baseline (BSL) and 5 (P05), 15 (P15), 30 (P30), 45 (P45), and 60 (P60) minutes after exercise cessation. Error bars indicate the 95% confidence interval. ^∗^Indicates a significant difference from BSL; *p*-value < 0.05.

There was a significant interaction 6(*TIME*) × 4(*MAP*) for the microstate mean duration [*F*(15,555) = 3.79, *p* < 0.001]. *Post hoc* analyses revealed that the mean duration of map C was significantly increased (+8.7%) by exercise and remained higher compared to BSL during all recovery time points (all *p* < 0.001). In P60, the map C duration had decreased (-3.4%) from P05 (*p* = 0.007), but did not return to BSL (*p* < 0.001) suggesting an uncomplete recovery. A short-term increase (+4.2%) in the map B duration was also found in P05 (*p* = 0.04) and returned to the pre-exercise value in P15 (*p* < 0.05) (**Figure 4A**).

There was a significant 6(*TIME*) × 4(*MAP*) interaction for the microstate time coverage [*F*(15,555) = 3.7, *p* < 0.001]. *Post hoc* tests demonstrated an increase in the time coverage only for map C in P05 (*p* < 0.001), P15 (*p* = 0.03), and P30 (*p <* 0.002) when compared to BSL. In P45 and P60 the time coverage tended to be different from BSL (*p* = 0.07 and *p* = 0.08, respectively) and thus indicated a return to the pre-exercise value (**Figure 4B**).

A significant 6(*TIME*) × 4(*MAP*) interaction was also found for the frequency of occurrence [*F*(15,555) = 1.99, *p* = 0.014]. Bonferroni *post hoc* tests indicated a significant change for map D immediately after exercise in P05 (*p* = 0.005). At P15 and subsequent time points, the map D occurrence was not different from BSL (**Figure 4C**).

**FIGURE 5:**
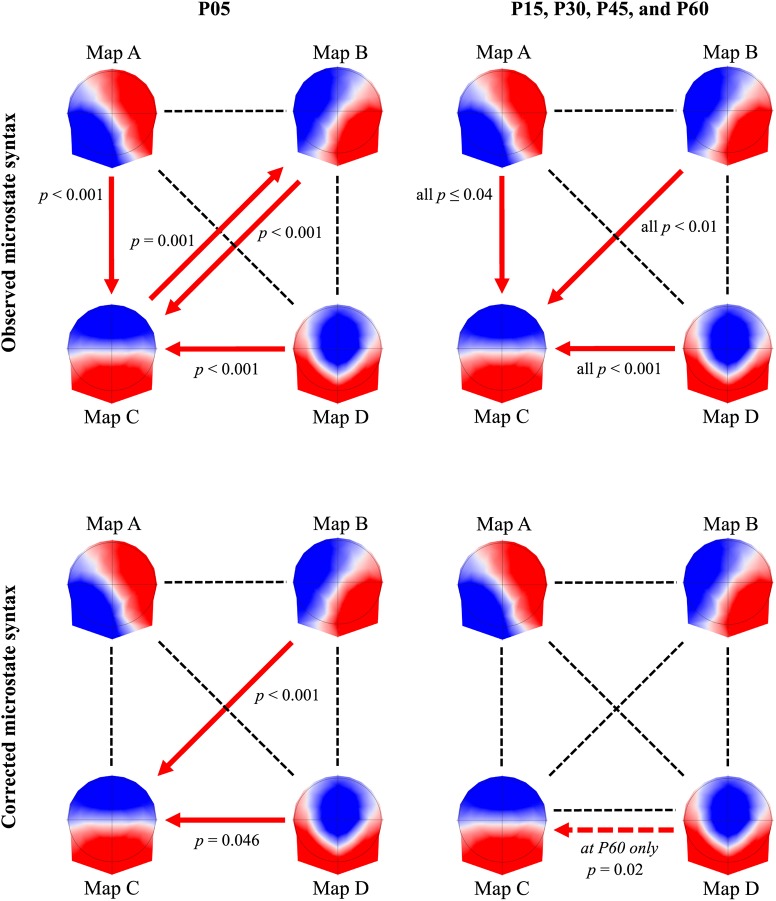
Microstate syntax. Microstate syntax changes from baseline, reported 5 min (P05) after exercise cessation (first column), and from 15 min (P15) to 60 min (P60) post-exercise (second column). The first row shows the observed microstate syntax between the four microstates, whereas the second row shows the microstate syntax independent for changes in microstate occurrence. The red arrows indicate the direction of the significant changes. Note that the dotted red arrow indicates a significant difference in P60 condition only.

The rmANOVA showed a significant 6(*TIME*) × 12(*PAIRS*) interaction for the observed microstate syntax [*F*(55,2035) = 2.86, *p* < 0.001] (**Figure 5**). Compared to BSL, the probability of transition between A-C, B-C, C-B, and D-C was higher in P05 (all *p* ≤ 0.001). During the 60 min post-exercise, the transitions between A-C, B-C, and D-C remained significantly different from BSL (*p* ≤ 0.04) and the transition between B-C in P60 was different from P05 (*p* = 0.02). When the syntax was corrected for changes in occurrence, we still found a significant interaction [*F*(55,2035) = 1.69, *p* = 0.001]. The follow-up tests showed an increased transition between B-C (*p* < 0.001) and D-C (*p* = 0.046) in P05. In P60, the transition between D-C was also different from BSL (*p* = 0.02).

### HRV and EEG Correlations

**FIGURE 6:**
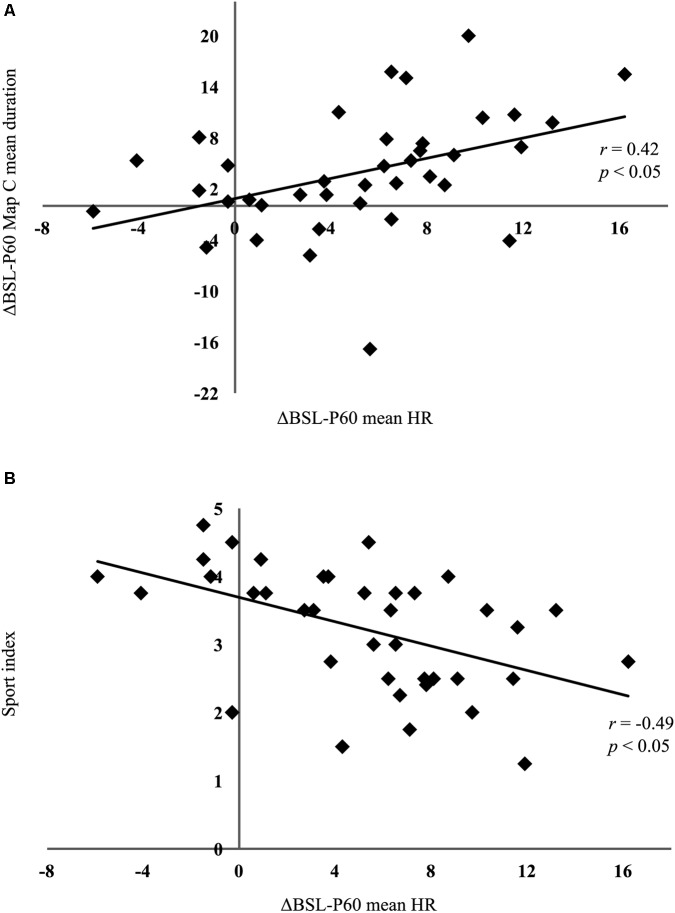
Correlation results. **(A)** Pearson correlation computed on the absolute differences (ΔBSL-P60) between baseline (BSL) and the post-60 min exercise condition (P60) for mean heart rate (HR) and microstate map C mean duration. The significant positive association indicates that the higher the difference in heart rate at P60, the longer the duration of map C remains. **(B)** Pearson correlation computed on the differences between BSL and P05 (ΔBSL-P05) for mean HR and the Sport index collected with the Baecke questionnaire. Note that people who have a lower Sport index are those who have a greater HR difference, and thus have less well recovered 60 min after the end of exercise.

Pearson correlations indicated that the exercise-induced microstate map C (mean duration, time coverage, and frequency of occurrence) and HRV parameters (mean HR, RMSSD, nLF, nHF, LF/HF ratio) changes were not correlated between BSL and P05(ΔBSL-P05), and between P05 and P60 (ΔP05-P60). However, between BSL and P60 (ΔBSL-P60), the microstate map C mean duration was significantly correlated with the ΔBSL-P60 mean HR (*r* = 0.42, *p* < 0.05) (**Figure 6A**). Because the HR recovery is related to the level of fitness, we decided to correlate *a posteriori* the ΔBSL-P60 mean HR with the Sport index collected with the Baecke questionnaire. We found a significant negative correlation (*r* = −0.49, *p* < 0.05), indicating that people who reported to be more active before starting the protocol are those who better recover (**Figure 6B**).

## Discussion

The present study aimed to describe the resting EEG microstate and HRV changes after a single-bout of physical exercise as well as the specific implication of the microstate map C after exercise. In accordance with a similar study conducted in trained male athletes (Spring et al., 2017), our results partly confirm that an acute submaximal endurance exercise of approximately 30 min modulates the microstate map C mean duration and time coverage, and is accompanied by an increased probability of transition mainly toward map C. The new outcome is that the microstate temporal change is not transient, but persists for at least 1 h after exercise cessation, suggesting a long-lasting effect of exercise on this specific microstate. In contrast to Spring et al. (2017), a short-term effect of exercise was found for the duration of map B and the frequency of occurrence of map D. According to the literature, the mean HR, RMSSD, LF, HF, nLH, nHF, and LF/HF ratio parameters were modulated by exercise. Except for the LF power, all the HRV indices of autonomic cardiovascular activity remained changed in the post-exercise period, indicating an incomplete recovery of the autonomic balance 1 h after exercise cessation. The correlation analyses performed on the delta between BSL and P60 showed a significant relationship between the microstate map C mean duration and mean HR, indicating that people who better recover in term of HR are also those who better recover in term of map C mean duration. In summary, when young adults performed a submaximal constant-load exercise of approximately 30 min, 1 h is not sufficient for both the autonomic cardiovascular activity and resting microstate to return to baseline.

### Microstate Data

Acute exercise increased the microstate map C duration and time coverage without modifying the frequency of occurrence in the immediate post-exercise period. These modulations are in line with the results reported by Spring et al. (2017). Similarly, the increased transition probability to move from map A, B, and D to the map C, initially described by Spring et al. (2017), was also found in the present study confirming that the map C becomes a predominant attractive microstate after exercise. During the hour following exercise cessation, even if the map C duration slightly decreased, the value was still significantly different from the pre-exercise condition, indicating that the microstate changes had not fully recovered.

During the immediate post-exercise measurement, we reported a transient increase in map B mean duration, a reduction in map D occurrence, and an increased transition from map B and D to the map C. This short-term microstate temporal reorganization in P05 was different from the long-lasting microstate configuration characterized by persistent changes in map C temporal properties only.

The literature on exercise and EEG microstates is almost non-existent, making difficult the interpretation of these findings. However, as microstate may correspond to particular classes of mentation, and perceptual and behavioral performances may vary as a function of ongoing microstate activity (Britz and Michel, 2011), we questioned if post-exercise brain modulations could comprise a kind of neural substrate underlying cognitive changes. In a similar exercise protocol consisting of 35 min of cycling at 90% of the ventilator threshold (i.e., at a HR between 142 to 152 beats/min and RPE between 12 and 15), Audiffren et al. (2008) reported changes in cognitive performance (i.e., information processing) only in the post-exercise period (between 1 and 6 min), but no effect after 15 min of recovery. Generally speaking, exercise of moderate intensity (40–80% VO_2max_) can have a positive effect on cognitive performance (Lambourne and Tomporowski, 2010; Chang et al., 2012; McMorris and Hale, 2015), including visual task performance (Lambourne and Tomporowski, 2010; Chang et al., 2012; Bullock and Giesbrecht, 2014). To explain this improvement, Bullock and Giesbrecht (2014) referred to the theory of visual attention (Bundesen et al., 2011), in which the size of the neural assemblies mobilized to encode a relevant object appear to be determinant. As microstate B has been associated with the visual RSN (Britz et al., 2010), the increase in the map B duration after exercise may reflect an increased stability within the related neural assemblies, proving supplementary brain resources that might be mobilized for a visual task. In the other hand, the better availability of attentional resources suggests a beneficial effect on cognition, and it has been hypothesized that the improvement in simple information processing after acute moderate exercise relies on increased arousal (McMorris and Graydon, 2000; Tomporowski, 2003; Davranche and Audiffren, 2004; McMorris et al., 2015). As microstate D has been associated with the dorsal attentional RSN and is likely to be involved in switching and reorientation of attention (Corbetta and Shulman, 2002; Britz et al., 2010), we thus questioned if modulation in map D occurrence might reflect modulations in complex switching attentional processes. Obviously, the above interpretation is merely speculative as no cognitive measurements have been undertaken. Nevertheless, it raises interesting perspectives about the brain mechanisms that may underlie post-exercise cognitive changes. Investigating the association between microstates and cognitive performance after exercise deserve to be explored.

### HRV Data

Sympathetic and parasympathetic HRV parameters were modulated immediately following exercise. RMSSD, HF, and nHF decreased, reflecting a parasympathetic withdrawal, whereas the increased in the LF/HF ratio indicated of a shift toward sympathetic overdrive (Malik, 1996). The normalized frequency power indices increased gradually during recovery but had not returned to BSL 1 h after exercise cessation. The amount of time required for the autonomic cardiovascular system to recover depends on exercise type (Esco et al., 2015), intensity and duration (Hautala et al., 2001; Seiler et al., 2007). In the present study, 25 min at an intensity perceived as “hard” resulted in modifications in the HRV that lasted at least 1 h. These findings agree with previous research conducted in young adults using a similar exercise protocol. Esco et al. (2015) found that after a cycling exercise of 30 min at slightly lower intensity (65% VO_2_ reserve), HRV parameters (nHF, nLF, LH/HF) were still different from baseline 30 min after exercise cessation. Terziotti et al. (2001) implemented a steady-state exercise of 20 min on a cycle ergometer at 50 or 80% of the anaerobic threshold and measured the HRV parameters up to 180 min after exercise. After 60 min, even if some subjects displayed signs of sympathetic activation, no significant differences were observed in the HRV parameters compared to baseline. The authors concluded that 1 h of rest is sufficient to fully recover after an exercise bout of 20 min at 50% of the anaerobic threshold, and just sufficient to recover after an exercise bout at 80% of the anaerobic threshold. In our study, the intensity was similar to the 80% condition, but the duration was longer, which certainly extended the time required to fully recover. The HF and LF powers were lower after exercise with an imbalance toward sympathetic activity as evidenced by the greater reduction in HF compared to LF, and by the increase in LF/HF ratio. The sympathetic withdrawal contributes to HR recovery soon after exercise as reflected by LF power that returned to BSL 30 min after the end of exercise. A long-lasting effect was found for the vagal modulation of HR as evidenced by the persistent changes in HF power during the whole recovery period. As almost all HRV indices of the autonomic cardiac activity remained significantly different from pre-exercise values during the entire recovery period, we infer that both autonomic nervous system components were responsible for the persistent changes in HR.

### Microstate and HRV Correlations

In contrast to resting EEG studies showing an association between the electrocortical signal and a specific component of the autonomic cardiovascular system (Duschek et al., 2015; Triggiani et al., 2016), the microstate parameters changes after exercise (ΔBSL-P05) and during recovery (ΔP05-P60) were not correlated with HRV. Duschek et al. (2015) reported a significant correlation between the R-wave-to-pulse interval, which is thought to reflect the sympathetic control of HR, and frontal beta power (13–30 Hz). The correlation was rather weak (*r* = −0.28, *p* < 0.05) and no correlations were found between the EEG power and the respiratory sinus arrhythmia (RSA, variation in R-R interval during the breathing cycle) or the baroreflex sensitivity, two other parameters reflecting the cardiovascular control. Using EEG source localization method, Triggiani et al. (2016) found a negative correlation (*r* = −0.42, *p* < 0.05) between the resting central Rolandic cortical source in the low-frequency beta band (13–20 Hz) and the LF power, suggesting that Rolandic beta rhythms are related to sympathetic activity. In an interventional task, Chang et al. (2011) investigated the relationships between changes in the spectral EEG and the HRV when the body position is changed from the supine to the upright position. When passing from the supine to the upright position, the theta rhythms (0.4–8 Hz) were associated with the HR, the alpha (8–14 Hz) and beta (14–35 Hz) bands with the vagal modulation, and the gamma band (35–45 Hz) with the sympathetic modulation. Even if an association between the electrocortical activity and some cardiovascular parameters can be identified at rest, this relationship seems to be tenuous, especially after physical exercise. The variability of the exercise-induced physiological response and the different temporal fluctuation of the heart and the brain activities probably make this interaction difficult to observe.

Interestingly, the microstate C duration and almost all HRV parameters remained higher 1 h after exercise cessation, confirming that both the autonomic cardiac activity and the resting EEG microstate did not fully recover. Furthermore, a significant correlation was found on ΔBSL-P60 between the mean HR and microstate map C mean duration. After 1 h of passive recovery, the higher the difference in HR, the greater the difference of microstate map C mean duration. This correlation is in accordance with the functional association between the HRV and the main brain nodes of the salience network. Several neuroimaging studies have related HRV with fluctuations in brain connectivity within different neural structures including the insula and cingulate (Critchley et al., 2003; Napadow et al., 2008; Lane et al., 2009; Thayer et al., 2012). For instance, Chang et al. (2013) described covariations between resting HRV and several brain regions, including the dorsal anterior cingulate cortex and anterior insula, whereas Critchley et al. (2003) reported that the dorsal anterior cingulate is involved in autonomic control during cognitive processing, but also during a motor task consisting of isometric handgrip contractions. Consequently, we infer that the post-exercise microstate C and mean HR recovery may rely on the functional connection between the salience RSN and the autonomic cardiovascular system.

The post-exercise autonomic cardiovascular reactivation is thought to largely depends on accumulation of stress metabolites (e.g., H^+^, lactate) likely driven by metaboreceptor feedbacks (Boushel, 2010; Coote, 2010; Hartwich et al., 2011; Stanley et al., 2013; Peçanha et al., 2014, 2016). The reflex inputs from metaboreceptors and mechanorecptors in the active muscles has been postulated as an important mechanism in cardiovascular and ventilatory regulation (Kaufman and Hayes, 2002; Secher and Amann, 2012; Fisher et al., 2013). The mechanical and chemical stimuli associated with muscle contraction activate the terminal ends of small-diameter type III and IV afferent fibers that stimulate the lamina I neurons within the spinal cord. From the superficial layer of the dorsal spinal horn, an afferent pathway caries information through the lateral spinothalamic tract to the thalamus (Craig, 2002, 2003), where projections relay the information from thalamic nuclei to the mid/posterior dorsal insula that in turn project to AI (Menon and Uddin, 2010). The implication of this feedback was supported by several experimental manipulations. For instance, the lowering of the afferent feedback during exercise by a pharmacological blockade attenuate the increase in blood pressure and cardiac output (Secher and Amann, 2012). In post-exercise condition, when a blood flow restriction is applied in order to occlude the blood supply and to trap the metabolites in the active muscle (i.e., post-exercise ischemia), the exercise-induced increase in HR (Fisher et al., 2013; Macefield and Henderson, 2015), sympathetic activity (Victor and Seals, 1989), and/or withdrawal of cardiac parasympathetic tone (Fisher et al., 2013) do not return to baseline. Furthermore, the autonomic response to post-exercise ischemia indicates an increase in blood-oxygen-level dependent signal within the insular cortex, and a contribution of an afferent feedback associated with the exercise-induced physiological changes has been postulated (Sander et al., 2010; Macefield and Henderson, 2015). Although the intramuscular metabolites have not been quantified in the present article, previous study reported higher circulating lactate value 50 min after eight, 5-min bouts of cycling at 80% of maximum workload, suggesting long-lasting metabolic disturbance (Sidhu et al., 2008). Yet, depending on the blood acidosis and blood lactate level, the post-exercise HR recovery would be delayed (Ba et al., 2009). Taken together, modifications in afferent activity after exercise may participate in modulating the autonomic cardiovascular response and recovery, and potentially the microstate map C temporal properties.

Time series analysis allows the exploration of the dynamical interactions between physiological systems. Recent advanced signal processing tools, such as multivariate method based on Granger causality, investigate the neural and cardiac time series and demonstrate a functional coupling between the brain and the heart (Yu et al., 2014; Porta and Faes, 2016). In particular, reciprocal influence between cardiac component and the EEG power has been reported (Faes et al., 2014). An interesting methodological perspective would be to consider the microstate analysis as an alternative signal processing technique that may provide a new approach for investigating brain-heart interactions.

### Limitations

Investigating HRV spectral indices of the autonomic cardiovascular activity during the post-exercise recovery assume stationarity of the signal. However, in some cases the results from the Likelihood ratio test indicate slow drift of the HRV signal. We have to consider that the non-stationarity of our data could distort the short-term spectral HRV indices and may contribute to an overestimation of the sympathetic control (Magagnin et al., 2011). However, the characteristics of non-stationary reflect the equilibrium control activities within autonomic nervous system and may contain valuable information for autonomic nervous system assessment (Ding et al., 2007). Moreover, the scale-free dynamics of microstate sequences itself imply non-stationarity (Van De Ville et al., 2010). Applying a detrending method on the present dataset will destroy the temporal dynamic property of the signals, which is an integral part of the microstate sequence and the recovery process. It is important to note that the indices used in the present study reflect an estimation that simplifies the physiological complexity of the autonomic control and brain activity recovery, and should be then taken with caution.

## Conclusion

The present study investigated the resting EEG microstates and HRV before and during 1 h after a constant-load cycling exercise perceived as “hard” in healthy males and females. Acute exercise modulates the microstate C duration with an effect that persists for at least 1 h after exercise, suggesting that this specific topography may be considered as an electrocortical index of exercise-related brain modulation. As for the duration of map C, the markers of cardiac autonomic activity (mean HR, RMSSD, nHF, nLF) were modulated immediately after exercise and for 1 h post-exercise. The HR recovery correlates with the recovery of microstate C duration, indicating a possible interaction between the cardiovascular system and microstate map C. The functional association between HRV and salience network brain nodes reported in the literature (Thayer et al., 2012) further support our finding. We assumed that the microstate C and autonomic cardiovascular activity changes are likely mediated by a common increase in exercise-induced afferent activity. The metabolic byproducts of fatiguing muscular exercise stimulates the metaboreceptor afferents and may modulate the autonomic regulation of HR after exercise. As the autonomic and muscle afferents seems to converge to the salience RNS nodes (Craig, 2003; Menon, 2015), an increase in afferent neural traffic after exercise might be considered as a mediator of both the autonomic regulation of HR and the resting EEG microstate.

## Ethics Statement

This study was carried out in accordance with the recommendations of the Ethics committee Vaud (CER-VD: 2016-01730) with written informed consent from all subjects. All subjects gave written informed consent in accordance with the Declaration of Helsinki. The protocol was approved by the CER-VD.

## Author Contributions

JS and JB conceived, planned the experiment, and carried out the data collection. JS, NB, and JB participated in analyzing the data and contributed to the interpretation. JB was in charge of overall direction.

## Conflict of Interest Statement

The authors declare that the research was conducted in the absence of any commercial or financial relationships that could be construed as a potential conflict of interest.

## Footnotes

1 brainmapping.unige.ch/cartool

## References

[B1] Audiffren M. Tomporowski P. D. Zagrodnik J. (2008). Acute aerobic exercise and information processing: energizing motor processes during a choice reaction time task. *Acta Psychol.* 129 410–419. 10.1016/j.actpsy.2008.09.006 18930445

[B2] Ba A. Delliaux S. Bregeon F. Levy S. Jammes Y. (2009). Post-Exercise heart rate recovery in healthy, Obeses, and COPD subjects: relationships with blood lactic acid and PaO2 Levels. *Clin. Res. Cardiol.* 98 52–58. 10.1007/s00392-008-0723-0 18853089

[B3] Baecke J. A. Burema J. Frijters J. E. (1982). A short questionnaire for the measurement of habitual physical activity in epidemiological studies. *Am. J. Clin. Nutr.* 36 936–942. 10.1093/ajcn/36.5.936 7137077

[B4] Bechara A. Naqvi N. (2004). Listening to your heart: interoceptive awareness as a gateway to feeling. *Nat. Neurosci.* 7 102–103. 10.1038/nn0204-102 14747831

[B5] Boushel R. (2010). Muscle metaboreflex control of the circulation during exercise. *Acta Physiol.* 199 367–383. 10.1111/j.1748-1716.2010.02133.x 20353495

[B6] Britz J. Michel C. M. (2011). State-dependent visual processing. *Front. Psychol.* 2:370. 10.3389/fpsyg.2011.00370 22203809 PMC3241342

[B7] Britz J. Van De Ville D. Michel C. M. (2010). BOLD Correlates of EEG topography reveal rapid resting-state network dynamics. *Neuroimage* 52 1162–1170. 10.1016/j.neuroimage.2010.02.052 20188188

[B8] Brunet D. Murray M. M. Michel C. M. (2011). Spatiotemporal analysis of multichannel EEG: CARTOOL. *Comput. Intell. Neurosci.* 2011:813870. 10.1155/2011/813870 21253358 PMC3022183

[B9] Bullock T. Giesbrecht B. (2014). Acute exercise and aerobic fitness influence selective attention during visual search. *Front. Psychol.* 5:1290. 10.3389/fpsyg.2014.01290 25426094 PMC4227487

[B10] Bundesen C. Habekost T. Kyllingsbaek S. (2011). A neural theory of visual attention and short-term memory (NTVA). *Neuropsychologia* 49 1446–1457. 10.1016/j.neuropsychologia.2010.12.006 21146554

[B11] Chang C. Metzger C. D. Glover G. H. Duyn J. H. Heinze H. J. Walter M. (2013). Association between heart rate variability and fluctuations in resting-state functional connectivity. *Neuroimage* 68 93–104. 10.1016/j.neuroimage.2012.11.038 23246859 PMC3746190

[B12] Chang L.-J. Lin J.-F. Lin C. F. Wu K.-T. Wang Y.-M. Kuo C.-D. (2011). Effect of body position on bilateral EEG alterations and their relationship with autonomic nervous modulation in normal subjects. *Neurosci. Lett.* 490 96–100. 10.1016/j.neulet.2010.12.034 21182897

[B13] Chang Y. K. Labban J. D. Gapin J. I. Etnier J. L. (2012). The effects of acute exercise on cognitive performance: a meta-analysis. *Brain Res.* 1453 87–101. 10.1016/j.brainres.2012.02.068 22480735

[B14] Christensen N. J. Galbo H. (1983). Sympathetic nervous activity during exercise. *Annu. Rev. Physiol.* 45 139–153. 10.1146/annurev.ph.45.030183.001035 6342511

[B15] Coote J. H. (2010). Recovery of heart rate following intense dynamic exercise. *Exp. Physiol.* 95 431–440. 10.1113/expphysiol.2009.047548 19837772

[B16] Corbetta M. Shulman G. L. (2002). Control of goal-directed and stimulus-driven attention in the brain. *Nat. Rev. Neurosci.* 3 201–215. 10.1038/nrn755 11994752

[B17] Craig A. D. (1995). Distribution of brainstem projections from spinal lamina I neurons in the cat and the monkey. *J. Comp. Neurol.* 361 225–248. 10.1002/cne.903610204 8543660

[B18] Craig A. D. (2002). How do you feel? Interoception: the sense of the physiological condition of the body. *Nat. Rev. Neurosci.* 3 655–666. 10.1038/nrn894 12154366

[B19] Craig A. D. (2003). Interoception: the sense of the physiological condition of the body. *Curr. Opin. Neurobiol.* 13 500–505. 12965300 10.1016/s0959-4388(03)00090-4

[B20] Critchley H. D. Corfield D. R. Chandler M. P. Mathias C. J. Dolan R. J. (2000). Cerebral correlates of autonomic cardiovascular arousal: a functional neuroimaging investigation in humans. *J. Physiol.* 523 259–270. 10.1111/j.1469-7793.2000.t01-1-00259.x 10673560 PMC2269796

[B21] Critchley H. D. Mathias C. J. Josephs O. O’Doherty J. Zanini S. Dewar B. K. (2003). Human cingulate cortex and autonomic control: converging neuroimaging and clinical evidence. *Brain* 126 2139–2152. 10.1093/brain/awg216 12821513

[B22] Darr K. C. Bassett D. R. Morgan B. J. Thomas D. P. (1988). Effects of age and training status on heart rate recovery after peak exercise. *Am. J. Physiol. Heart Circ. Physiol.* 254 H340–H343. 10.1152/ajpheart.1988.254.2.H340 3344824

[B23] Davranche K. Audiffren M. (2004). Facilitating effects of exercise on information processing. *J. Sports Sci.* 22 419–428. 10.1080/02640410410001675289 15160595

[B24] Ding H. Crozier S. Wilson S. (2007). A New heart rate variability analysis method by means of quantifying the variation of nonlinear dynamic patterns. *IEEE Trans. Biomed. Eng.* 54 1590–1597. 10.1109/TBME.2007.893495 17867351

[B25] Duschek S. Wörsching J. Reyes del Paso G. A. (2015). Autonomic cardiovascular regulation and cortical tone. *Clin. Physiol. Funct. Imaging* 35 383–392. 10.1111/cpf.12174 25080269

[B26] Eckert M. A. Menon V. Walczak A. Ahlstrom J. Denslow S. Horwitz A. (2009). At the heart of the ventral attention system: the right anterior insula. *Hum. Brain Mapp.* 30 2530–2541. 10.1002/hbm.20688 19072895 PMC2712290

[B27] Esco M. R. Flatt A. A. Williford H. N. (2015). Postexercise heart rate variability following treadmill and cycle exercise: a comparison study. *Clin. Physiol. Funct. Imaging* 37 322–327. 10.1111/cpf.12308 26442473

[B28] Faes L. Nollo G. Jurysta G. Marinazzo G. (2014). Information dynamics of brain–heart physiological networks during sleep. *New J. Phys.* 16:105005 10.1088/1367-2630/16/10/105005

[B29] Fisher J. P. Adlan A. M. Shantsila A. Frederik Secher J. Sørensen H. Secher N. H. (2013). Muscle metaboreflex and autonomic regulation of heart rate in humans. *J. Physiol.* 591 3777–3788. 10.1113/jphysiol.2013.254722 23713032 PMC3752457

[B30] Furlan R. Piazza S. Dell’Orto S. Gentile E. Cerutti S. Pagani M. (1993). Early and late effects of exercise and athletic training on neural mechanisms controlling heart rate. *Cardiovasc. Res.* 27 482–488. 10.1093/cvr/27.3.482 8490949

[B31] Gärtner M. Brodbeck V. Laufs H. Schneider G. (2015). A stochastic model for EEG microstate sequence analysis. *Neuroimage* 104(Suppl. C), 199–208. 10.1016/j.neuroimage.2014.10.014 25451473

[B32] Hartwich D. Dear W. E. Waterfall J. L. Fisher J. P. (2011). Effect of muscle metaboreflex activation on spontaneous cardiac baroreflex sensitivity during exercise in humans. *J. Physiol.* 589 6157–6171. 10.1113/jphysiol.2011.219964 21969452 PMC3286693

[B33] Hautala A. Tulppo M. P. Mäkikallio T. H. Laukkanen R. Nissilä S. Huikuri H. V. (2001). Changes in cardiac autonomic regulation after prolonged maximal exercise. *Clin. Physiol.* 21 238–245. 10.1046/j.1365-2281.2001.00309.x 11318832

[B34] Hill D. W. Cureton K. J. Grisham S. C. Collins M. A. (1987). Effect of training on the rating of perceived exertion at the ventilatory threshold. *Eur. J. Appl. Physiol. Occup. Physiol.* 56 206–211. 10.1007/BF00640645 3569227

[B35] Jung T.-P. Makeig S. Westerfield M. Townsend J. Courchesne E. Sejnowski T. J. (2000). Removal of eye activity artifacts from visual event-related potentials in normal and clinical subjects. *Clin. Neurophysiol.* 111 1745–1758. 10.1016/S1388-2457(00)00386-2 11018488

[B36] Kaufman M. P. Hayes S. G. (2002). The exercise pressor reflex. *Clin. Auton. Res.* 12 429–439. 10.1007/s10286-002-0059-1 12598947

[B37] Khanna A. Pascual-Leone A. Michel C. M. Farzan F. (2015). Microstates in resting-State EEG: current status and future directions. *Neurosci. Biobehav. Rev.* 49 105–113. 10.1016/j.neubiorev.2014.12.010 25526823 PMC4305485

[B38] Koenig T. Lehmann D. Merlo M. C. Kochi K. Hell D. Koukkou M. (1999). A deviant EEG brain microstate in acute, neuroleptic-naive schizophrenics at rest. *Eur. Arch. Psychiatry Clin. Neurosci.* 249 205–11. 10.1007/s004060050088 10449596

[B39] Koenig T. Prichep L. Lehmann D. Sosa P. V. Braeker E. Kleinlogel H. (2002). Millisecond by millisecond, year by year: normative EEG microstates and developmental stages. *Neuroimage* 16 41–48. 10.1006/nimg.2002.1070 11969316

[B40] Lambourne K. Tomporowski P. (2010). The effect of exercise-induced arousal on cognitive task performance: a meta-regression analysis. *Brain Res.* 1341 12–24. 10.1016/j.brainres.2010.03.091 20381468

[B41] Lane R. D. McRae K. Reiman E. M. Chen K. Ahern G. L. Thayer J. F. (2009). Neural correlates of heart rate variability during emotion. *Neuroimage* 44 213–222. 10.1016/j.neuroimage.2008.07.056 18778779

[B42] Lehmann D. Faber P. L. Galderisi S. Herrmann W. M. Kinoshita T. Koukkou M. (2005). EEG microstate duration and syntax in acute, medication-naïve, first-episode schizophrenia: a multi-center study. *Psychiatry Res.* 138 141–156. 10.1016/j.pscychresns.2004.05.007 15766637

[B43] Lehmann D. Pascual-Marqui R. Michel M. (2009). EEG Microstates. *Scholarpedia* 4:7632 10.4249/scholarpedia.7632

[B44] Macefield V. G. Henderson L. A. (2015). Autonomic responses to exercise: cortical and subcortical responses during post-exercise ischaemia and muscle pain. *Auton. Neurosci.* 188 10–18. 10.1016/j.autneu.2014.10.021 25458426

[B45] Magagnin V. Bassani T. Bari V. Turiel M. Maestri R. Pinna G. D. (2011). Non-stationarities significantly distort short-term spectral, symbolic and entropy heart rate variability indices. *Physiol. Meas.* 32 1775–1786. 10.1088/0967-3334/32/11/S05 22027399

[B46] Malik M. (1996). Heart rate variability standards of measurement, physiological interpretation, and clinical use. *Eur. Heart J.* 17 354–381. 10.1093/oxfordjournals.eurheartj.a014868 8737210

[B47] McMorris T. Graydon J. (2000). The effect of incremental exercise on cognitive performance. *Int. J. Sport Psychol.* 31 66–81.

[B48] McMorris T. Hale B. J. (2015). Is there an acute exercise-induced physiological/biochemical threshold which triggers increased speed of cognitive functioning? A meta-analytic investigation. *J. Sport Health Sci.* 4 4–13. 10.1016/j.jshs.2014.08.003

[B49] McMorris T. Hale B. J. Corbett J. Robertson K. Hodgson C. I. (2015). Does acute exercise affect the performance of whole-body, psychomotor skills in an inverted-u fashion? A meta-analytic investigation. *Physiol. Behav.* 141 180–189. 10.1016/j.physbeh.2015.01.010 25582516

[B50] Menon V. (2015). “Salience network,” in *Brain Mapping*, ed. Toga W. (Waltham, MA: Academic Press), 597–611. 10.1016/B978-0-12-397025-1.00052-X

[B51] Menon V. Uddin L. Q. (2010). Saliency, switching, attention and control: a network model of insula function. *Brain Struct. Funct.* 214 655–667. 10.1007/s00429-010-0262-0 20512370 PMC2899886

[B52] Michel C. M. Koenig T. (2017). EEG microstates as a tool for studying the temporal dynamics of whole-brain neuronal networks: a review. *NeuroImage* 10.1016/j.neuroimage.2017.11.062 [Epub ahead of print]. 29196270

[B53] Michel C. M. Koenig T. Brandeis D. Gianotti L. R. R. Wackermann J. (2009). *Electrical Neuroimaging.* Cambridge: Cambridge University Press. 10.1017/CBO9780511596889

[B54] Mourot L. Bouhaddi M. Tordi N. Rouillon J.-D. Regnard J. (2004). Short- and long-term effects of a single bout of exercise on heart rate variability: comparison between constant and interval training. *Eur. J. Appl. Physiol.* 92 508–517. 10.1007/s00421-004-1119-0 15461995

[B55] Murphy M. N. Mizuno M. Mitchell J. H. Smith S. A. (2011). Cardiovascular regulation by skeletal muscle reflexes in health and disease. *Am. J. Physiol. Heart Circ. Physiol.* 301 1191–1204. 10.1152/ajpheart.00208.2011 21841019 PMC3197431

[B56] Napadow V. Dhond R. Conti G. Makris N. Brown E. Barbieri R. (2008). Brain correlates of autonomic modulation: combining heart rate variability with fMRI. *Neuroimage* 42 169–177. 10.1016/j.neuroimage.2008.04.238 18524629 PMC2603289

[B57] Peçanha T. de Brito L. C. Fecchio R. Y. de Sousa P. N. da Silva Junior N. D. de Abreu A. P. (2016). Metaboreflex activation delays heart rate recovery after aerobic exercise in never-treated hypertensive men. *J. Physiol.* 594 6211–6223. 10.1113/JP272851 27435799 PMC5088244

[B58] Peçanha T. Silva-Júnior N. D. de Moraes Forjaz C. L. (2014). Heart rate recovery: autonomic determinants, methods of assessment and association with mortality and cardiovascular diseases. *Clin. Physiol. Funct. Imaging* 34 327–339. 10.1111/cpf.12102 24237859

[B59] Perini R. Orizio C. Comandè A. Castellano M. Beschi M. Veicsteinas A. (1989). Plasma norepinephrine and heart rate dynamics during recovery from submaximal exercise in man. *Eur. J. Appl. Physiol. Occup. Physiol.* 58 879–883. 10.1007/BF02332222 2767070

[B60] Perkins S. E. Jelinek H. F. Al-Aubaidy H. A. de Jong B. (2017). Immediate and long term effects of endurance and high intensity interval exercise on linear and nonlinear heart rate variability. *J. Sci. Med. Sport* 20 312–316. 10.1016/j.jsams.2016.08.009 27568073

[B61] Pollatos O. Schandry R. Auer D. P. Kaufmann C. (2007). Brain structures mediating cardiovascular arousal and interoceptive awareness. *Brain Res.* 1141 178–187. 10.1016/j.brainres.2007.01.026 17296169

[B62] Porta A. Faes L. (2016). Wiener–granger causality in network physiology with applications to cardiovascular control and neuroscience. *Proc. IEEE* 104 282–309. 10.1109/JPROC.2015.2476824

[B63] Purvis J. W. Cukiton K. J. (1981). Ratings of perceived exertion at the anaerobic threshold. *Ergonomics* 24 295–300. 10.1080/00140138108924852 7238495

[B64] Sander M. Macefield V. G. Henderson L. A. (2010). Cortical and brain stem changes in neural activity during static handgrip and postexercise ischemia in humans. *J. Appl. Physiol.* 108 1691–1700. 10.1152/japplphysiol.91539.2008 20185626

[B65] Savin W. M. Davidson D. M. Haskell W. L. (1982). Autonomic contribution to heart rate recovery from exercise in humans. *J. Appl. Physiol.* 53 1572–1575. 10.1152/jappl.1982.53.6.1572 7153152

[B66] Secher N. H. Amann M. (2012). Human investigations into the exercise pressor reflex. *Exp. Physiol.* 97 59–69. 10.1113/expphysiol.2011.057679 22125307 PMC12820754

[B67] Seiler S. Haugen O. Kuffel E. (2007). Autonomic recovery after exercise in trained athletes: intensity and duration effects. *Med. Sci. Sports Exerc.* 39 1366–1373. 10.1249/mss.0b013e318060f17d 17762370

[B68] Sidhu S. K. Bentley D. J. Carroll T. J. (2008). Locomotor exercise induces long-lasting impairments in the capacity of the human motor cortex to voluntarily activate knee extensor muscles. *J. Appl. Physiol.* 106 556–565. 10.1152/japplphysiol.90911.2008 19056999

[B69] Singer T. Critchley H. D. Preuschoff K. (2009). A common role of insula in feelings, empathy and uncertainty. *Trends Cogn. Sci.* 13 334–340. 10.1016/j.tics.2009.05.001 19643659

[B70] Skrandies W. (1990). Global field power and topographic similarity. *Brain Topogr.* 3 137–141. 10.1007/BF01128870 2094301

[B71] Spring J. N. Tomescu M. I. Barral J. (2017). A single-bout of endurance exercise modulates EEG microstates temporal features. *Brain Topogr.* 30 461–472. 10.1007/s10548-017-0570-2 28528447

[B72] Stanley J. Peake J. M. Buchheit M. (2013). Cardiac parasympathetic reactivation following exercise: implications for training prescription. *Sports Med.* 43 1259–1277. 10.1007/s40279-013-0083-4 23912805

[B73] Terziotti P. Schena F. Gulli G. Cevese A. (2001). Post-exercise recovery of autonomic cardiovascular control: a study by spectrum and cross-spectrum analysis in humans. *Eur. J. Appl. Physiol.* 84 187–194. 10.1007/s004210170003 11320634

[B74] Thayer F. J. Åhs F. Fredrikson M. Sollers J. J. Wager T. D. (2012). A meta-analysis of heart rate variability and neuroimaging studies: implications for heart rate variability as a marker of stress and health. *Neurosci. Biobehav. Rev.* 36 747–756. 10.1016/j.neubiorev.2011.11.009 22178086

[B75] Tomescu M. I. Rihs T. A. Becker R. Britz J. Custo A. Grouiller F. (2014). Deviant Dynamics of EEG Resting State Pattern in 22q11.2 deletion syndrome adolescents: a vulnerability marker of schizophrenia. *Schizophr. Res.* 157 175–181. 10.1016/j.schres.2014.05.036 24962438

[B76] Tomporowski P. D. (2003). Effects of acute bouts of exercise on cognition. *Acta Psychol.* 112 297–324. 10.1016/S0001-6918(02)00134-8 12595152

[B77] Triggiani A. I. Valenzano A. Del Percio C. Marzano N. Soricelli A. Petito A. (2016). Resting state Rolandic mu rhythms are related to activity of sympathetic component of autonomic nervous system in healthy humans. *Int. J. Psychophysiol.* 103 79–87. 10.1016/j.ijpsycho.2015.02.009 25660308

[B78] Tulppo M. P. Makikallio T. H. Takala T. E. Seppanen T. Huikuri H. V. (1996). Quantitative beat-to-beat analysis of heart rate dynamics during exercise. *Am. J. Physiol. Heart Circ. Physiol.* 271 244–252. 10.1152/ajpheart.1996.271.1.H244 8760181

[B79] Van De Ville D. Britz J. Michel C. M. (2010). EEG microstate sequences in healthy humans at rest reveal scale-free dynamics. *Proc. Natl. Acad. Sci. U.S.A.* 107 18179–18184. 10.1073/pnas.1007841107 20921381 PMC2964192

[B80] Victor R. G. Seals D. R. (1989). Reflex stimulation of sympathetic outflow during rhythmic exercise in humans. *Am. J. Physiol. Heart Circ. Physiol.* 257 2017–2024. 10.1152/ajpheart.1989.257.6.H2017 2603985

[B81] Williamson J. W. (2015). Autonomic responses to exercise: where is central command. *Auton. Neurosci.* 188 3–4. 10.1016/j.autneu.2014.10.011 25458428

[B82] Williamson J. W. McColl R. Mathews D. (2003). Evidence for central command activation of the human insular cortex during exercise. *J. Appl. Physiol.* 94 1726–1734. 10.1152/japplphysiol.01152.2002 12533504

[B83] Yu X. L. Zhang C. Zhang J. (2014). Causal interactions between the cerebral cortex and the autonomic nervous system. *Sci. China Life Sci.* 57 532–538. 10.1007/s11427-014-4627-0 24691996

